# Dyslipidemia Increases the Risk of Incident Hypertension in a Large Taiwanese Population Follow-Up Study

**DOI:** 10.3390/nu14163277

**Published:** 2022-08-10

**Authors:** Yu-Hsuan Lin, Yi-Hsueh Liu, Da-Wei Wu, Ho-Ming Su, Szu-Chia Chen

**Affiliations:** 1 Department of Anesthesiology, Kaohsiung Chang Gung Memorial Hospital, Chang Gung University College of Medicine, Kaohsiung 833, Taiwan; 2 Department of Internal Medicine, Kaohsiung Municipal Siaogang Hospital, Kaohsiung Medical University, Kaohsiung 812, Taiwan; 3 Division of Cardiology, Department of Internal Medicine, Kaohsiung Medical University Hospital, Kaohsiung 807, Taiwan; 4 Division of Pulmonary and Critical Care Medicine, Department of Internal Medicine, Kaohsiung Medical University Hospital, Kaohsiung Medical University, Kaohsiung 807, Taiwan; 5 Division of Nephrology, Department of Internal Medicine, Kaohsiung Medical University Hospital, Kaohsiung Medical University, Kaohsiung 807, Taiwan; 6 Faculty of Medicine, College of Medicine, Kaohsiung Medical University, Kaohsiung 807, Taiwan; 7 Research Center for Precision Environmental Medicine, Kaohsiung Medical University, Kaohsiung 807, Taiwan

**Keywords:** lipid profile, incident hypertension, follow-up, Taiwan Biobank

## Abstract

Dyslipidemia is an important risk factor for hypertension and is strongly associated with an elevated risk of cardiovascular diseases including atherosclerosis and stroke. In this study, we investigated correlations between lipid profiles, including triglycerides, total cholesterol (Chol), high-and low-density lipoprotein cholesterol (HDL-C/LDL-C), and Chol/HDL-C, and baseline and incident hypertension. A total of 26,965 subjects with 4 years of follow-up data were enrolled from the Taiwan Biobank. In the cross-sectional cohort, associations between the prevalence of hypertension and lipid profiles were examined in all study participants (n = 26,965). In the longitudinal cohort, these associations were further assessed in the participants without baseline hypertension (n = 21,454). Multivariable analysis revealed that those in the second quartile (Q2) of triglycerides (compared to Q1; odds ratio (OR), 1.402; *p* < 0.001); Q3 of triglycerides (compared to Q1; OR, 1.365; *p* < 0.001); Q4 of triglycerides (compared to Q1; OR, 1.617; *p* < 0.001); Q3 of HDL-C (compared to Q1; OR, 0.886; *p* = 0.042); Q4 of HDL-C (compared to Q1; OR, 0.819; *p* = 0.002); Q2 of Chol/HDL-C (compared to Q1; OR, 1.144; *p* = 0.042); Q3 of Chol/HDL-C (compared to Q1; OR, 1.149; *p* = 0.034); and Q4 of Chol/HDL-C (compared to Q1; OR, 1.225; *p* = 0.002) were significantly associated with incident hypertension. In summary, high Chol/HDL-C, low HDL-C, and high triglycerides were associated with a higher risk of incident hypertension in the enrolled Taiwanese participants.

## 1. Introduction

The prevalence rates of hypertension in Taiwan are 18% in women and 25% in men, rising to 47% for people over 60 years of age [1]. The pathogenesis of primary hypertension is unclear; however, it is probably due to environmental and genetic factors which combine to affect cardiovascular and kidney structures and function [2]. Despite the uncertainty with regards to the pathogenesis, strong and independent risk factors have been associated with the onset of primary hypertension, such as older age [3], obesity [4], family history [5], race [6], a reduced number of nephrons [7], a high-sodium diet [8], excessive alcohol consumption, and physical inactivity [9,10]. Hypertension is an important source of disease burden worldwide [11], responsible for around 54% of strokes and 47% of coronary heart disease cases [12]. Therefore, identifying risk factors for hypertension is crucial to allow for the optimal management of patients and to avoid complications.

Dyslipidemia is defined as an increase in plasma low-density lipoprotein cholesterol (LDL-C), total cholesterol (Chol), triglycerides, or a combination of these, and it is known to be an important risk factor for ischemic heart diseases [13]. The World Health Organization estimated that the global prevalence of a high plasma Chol level in adults > 25 years of age was approximately 39% in 2008 [14]. In addition, elevated plasma LDL-C levels are associated with more than one-third of deaths from stroke or ischemic heart disease. Dyslipidemia is associated with the development of atherosclerosis, which is also an important risk factor for adverse cardiovascular outcomes [15]. Moreover, high levels of LDL-C and low levels of high-density lipoprotein cholesterol (HDL-C) have been associated with stroke and myocardial infarction [16,17].

Previous studies have reported interactions between blood lipids and blood pressure (BP) that may underlie the pathophysiological mechanisms shared by dyslipidemia and hypertension, such as endothelial dysfunction [18,19] and decreased arterial elasticity [20]. Previous studies have reported significant correlations between dyslipidemia and hypertension [21], while others have suggested a weak association [22], and others have not found an association [23]. However, these studies were limited by the sample size and differences in population and study design. Therefore, the aim of this large longitudinal study was to explore correlations between lipid profiles, including triglycerides, Chol, HDL-C, LDL-C, and Chol/HDL-C ratio, and incident and baseline hypertension in community-dwelling Taiwanese residents.

## 2. Materials and Methods

### 2.1. The Taiwan Biobank (TWB)

The TWB was established with the aim of recording lifestyle and genetic data of Taiwanese adults living in the community, and it is the most comprehensive biobank of its type in Taiwan [24,25]. The TWB was approved by the Ethics and Governance Council of the TWB and the Institutional Review Board (IRB) of Biomedical Science Research, Academia Sinica, Taiwan.

The inclusion criteria for the TWB are: (1) age 30–70 years and (2) no history of cancer. All participants provide written informed consent before they are enrolled, and then blood samples are drawn as part of a physical examination. Interviews between the participants and a TWB researcher are also conducted to collect information on lifestyle factors, dietary habits, and family medical history. BP is measured using a digital BP machine by trained members of staff. The patients are asked to avoid smoking, exercising, and drinking beverages containing caffeine at least 30 min prior to the initial BP reading. Each BP reading (both diastolic BP (DBP) and systolic BP (SBP)) is performed in triplicate with 1–2 min between readings, and the average value is used for analysis.

### 2.2. Laboratory, Medical, and Demographic Variables

Demographic data (age and sex); data on lifestyle factors (exercise (defined as ≥30 min of physical activity ≥3 times in 1 week) and consumption of tobacco and alcohol); medical history (hypertension and diabetes mellitus (DM)); and laboratory data (HDL-C, hemoglobin, fasting glucose, Chol, uric acid, triglycerides, and LDL-C) were collected at baseline. The Modification of Diet in Renal Disease study equation was used to calculate the estimated glomerular filtration rate (eGFR) [26].

### 2.3. Definition of Baseline and Incident Hypertension

Baseline hypertension was defined as BP ≥ 140/90 mmHg and a history of hypertension. The participants who denied a history of hypertension and whose BP was <140/90 mmHg were classified as being free of hypertension. The subsequent development of hypertension (BP ≥ 140/90 mmHg) during the study period was defined as incident hypertension.

### 2.4. Study Participants

**Figure 1:**
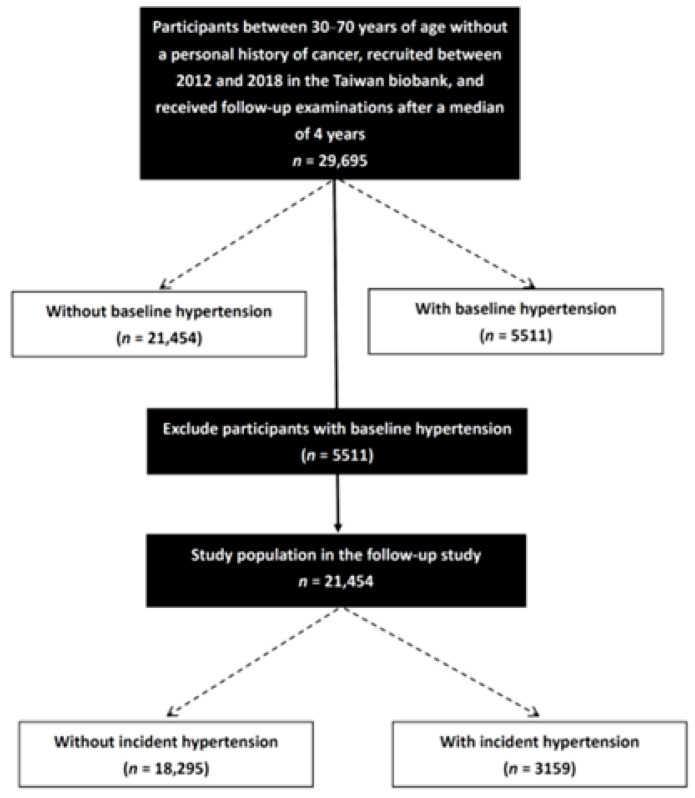
Study flowchart.

A total of 26,965 participants (9541 males; 17,424 females) were enrolled. The mean age of the participants was 51.2 ± 10.4 years, and the median follow-up period was 4 years. In the cross-sectional cohort, associations between the prevalence of hypertension and lipid profiles were assessed in all study participants (*n* = 26,965). The subjects with a history of hypertension (*n* = 5511) were excluded from the longitudinal cohort. The associations between incident hypertension and lipid profiles were further assessed in the subjects without baseline hypertension (*n* = 21,454) (Figure 1). This study was approved by the Institutional Review Board of Kaohsiung Medical University Hospital (KMUHIRB-E(I)-20210058), and it was conducted in accordance with the Declaration of Helsinki.

### 2.5. Statistical Analysis

The statistical analysis was performed using SPSS version 20.0 for Windows (SPSS Inc., Armonk, NY, USA). Categorical variables are shown as numbers (%), and the chi-square test was used to compare differences between groups. Continuous variables are shown as mean and standard deviation and were compared using the independent t-test. Associations between quartiles of lipid profiles with baseline and incident hypertension were evaluated using logistic regression analysis. The cutoff values of the quartiles were ≤63, 64–88, 89–127, and >127 mg/dL for triglycerides; ≤171, 172–193, 194–217, and >217 mg/dL for Chol; ≤46, 47–54, 55–63, and > 63 mg/dL for HDL-C; ≤99, 100–119, 120–141, and >141 mg/dL for LDL-C; and ≤2.96, 2.97–3.54, 3.55–4.29, and >4.29 for Chol/HDL-C ratio. The first quartiles (Q1) of HDL-C, Chol, LDL-C, Chol/HDL-C, and triglycerides were taken as reference values based on the lowest incidence. Multivariable analysis was conducted using the significant variables in univariable analysis. *p* values < 0.05 were considered statistically significant. 

## 3. Results

All enrolled participants were classified into two groups according to the presence (*n* = 5511; 20.4%) or absence (*n* = 21,454; 79.6%) of baseline hypertension.

### 3.1. Comparisons of Clinical Characteristics between Baseline Hypertension Groups

The baseline hypertension group were older and had more male participants than those without baseline hypertension (Table 1). In addition, they had higher rates of DM, smoking, alcohol use, and regular exercise, and higher levels of fasting glucose, hemoglobin, triglycerides, Chol, uric acid, Chol/HDL-C ratio, and LDL-C, but lower eGFR and HDL-C (Table 1).

We performed a further comparison of clinical characteristics according to sex in all study participants (Supplementary Table S1, *n* = 26,965). Compared to female participants (*n* = 17,424), male participants (*n* = 9541) were older and had higher prevalence rates of DM, baseline hypertension, smoking and alcohol history, and regular exercise; higher SBPl DBP, BMI, uric acid, fasting glucose, hemoglobin, Chol/HDL-C ratio, and triglycerides; and lower total cholesterol, HDL-C, and eGFR.

### 3.2. Associations between Lipid Profile Quartiles and Baseline Hypertension

**Figure 2:**
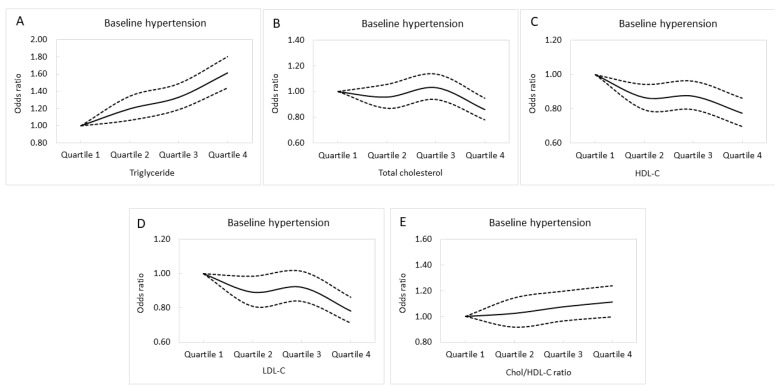
Adjusted baseline hypertension curves for the triglyceride (**A**), total cholesterol (**B**), HDL-C (**C**), LDL-C (**D**), and Chol/HDL-C (**E**) quartiles.

Multivariable logistic regression analysis of the whole cohort (*n* = 26,965) was used to investigate associations between lipid profile quartiles and baseline hypertension (Table 2). After adjusting for DM, the consumption of alcohol and tobacco, regular exercise, body mass index, uric acid, eGFR, fasting glucose, hemoglobin, sex, and age, those in the second quartile (Q2) of triglycerides (compared to Q1; odds ratio (OR), 1.197; *p* = 0.003); Q3 of triglycerides (compared to Q1; OR, 1.330; *p* < 0.001); Q4 of triglycerides (compared to Q1; OR, 1.612; *p* < 0.001); Q4 of Chol (compared to Q1; OR, 0.860; *p* = 0.002); Q2 of HDL-C (compared to Q1; OR, 0.866; *p* = 0.001); Q3 of HDL-C (compared to Q1; OR, 0.874; *p* = 0.006), Q4 of HDL-C (compared to Q1; OR, 0.774; *p* < 0.001), Q2 of LDL-C (compared to Q1; OR, 0.893; *p* = 0.023); and Q4 of LDL-C (compared to Q1; OR, 0.784; *p* < 0.001) were significantly associated with baseline hypertension. Adjusted baseline hypertension curves for the lipid profile quartiles are shown in Figure 2A–E.

In the follow-up study, after excluding the 5511 subjects who had histories of hypertension, 21,454 participants were included (6898 males, 14,556 females; mean age 49.7 ± 10.3 years). These participants were again divided according to the presence (*n* = 3159; 14.7%) or absence (*n* = 18,295; 85.3%) of incident hypertension.

### 3.3. Comparisons of Clinical Characteristics between Those with and without Incident Hypertension

The incident hypertension group were older and had more male participants than the group without incident hypertension (Table 3). In addition, they had higher rates of DM, tobacco and alcohol consumption, and regular exercise, and elevated levels of fasting glucose, hemoglobin, LDL-C, triglycerides, uric acid, Chol/HDL-C, and Chol, but lower HDL-C and eGFR (Table 3).

We further compared the clinical characteristics by sex in the study participants without baseline hypertension disease (Supplementary Table S2, *n* = 21,454). Compared to female participants (*n* = 14,556), male participants (*n* = 6898) had higher prevalence rates of DM and incident hypertension; higher tobacco and alcohol consumption, SBP, DBP, BMI, fasting glucose, LDL-C, uric acid, Chol/HDL-C, hemoglobin, and triglycerides; and lower total cholesterol, HDL-C, and eGFR.

### 3.4. Associations between Lipid Profile Quartiles and Incident Hypertension

**Figure 3:**
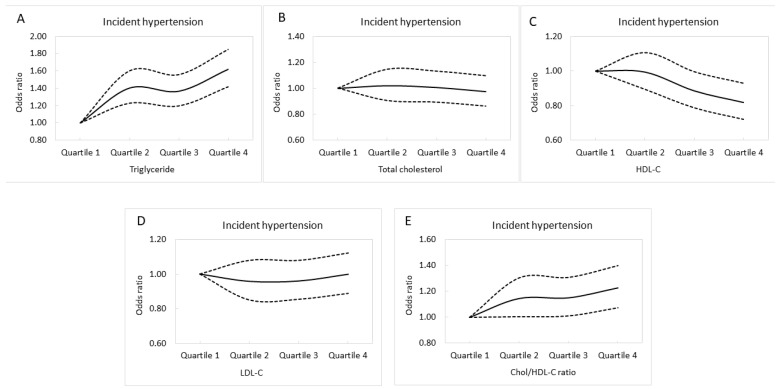
Adjusted incident hypertension curves for triglycerides (**A**), total cholesterol (**B**), HDL-C (**C**), LDL-C (**D**), and Chol/HDL-C (**E**) quartiles.

Multivariable logistic regression analysis of the follow-up cohort (*n* = 21,454) was performed to evaluate associations between incident hypertension and lipid profile quartiles (Table 4). After adjusting for age, sex, DM, the consumption of tobacco and alcohol, regular exercise, body mass index, hemoglobin, fasting glucose, eGFR, and uric acid, those in Q2 of triglycerides (compared to Q1; OR, 1.402; *p* < 0.001); Q3 of triglycerides (compared to Q1; OR, 1.365; *p* < 0.001); Q4 of triglycerides (compared to Q1; OR, 1.617; *p* < 0.001); Q3 of HDL-C (compared to Q1; OR, 0.886; *p* = 0.042); Q4 of HDL-C (compared to Q1; OR, 0.819; *p* = 0.002); Q2 of Chol/HDL-C ratio (compared to Q1; OR, 1.144; *p* = 0.042); Q3 of Chol/HDL-C (compared to Q1; OR, 1.149; *p* = 0.034); and Q4 of Chol/HDL-C (compared to Q1; OR, 1.225; *p* = 0.002) were significantly associated with incident hypertension. Adjusted incident hypertension curves for the lipid profile quartiles are shown in Figure 3A–E.

## 4. Discussion

In this study, we found associations between high triglycerides and low HDL-C and baseline hypertension and a higher risk of incident hypertension in our enrolled Taiwanese participants. Moreover, we also identified an association between high Chol/HDL-C and an increased risk of incident hypertension.

The association between a high triglyceride level and baseline hypertension and a higher risk of incident hypertension is an important finding. Participants in the second triglyceride quartile (64–88 mg/dL) exhibited a 1.402-times increased risk of incident hypertension. Previous cohort studies have reported an independent association between a higher concentration of serum triglycerides and the development of hypertension [27,28,29,30,31]. The results of our study are consistent with those of other studies that reported that hypertriglyceridemia is a risk factor for hypertension. The mechanism underlying the association between hypertriglyceridemia and an increase in BP is poorly understood. Some researchers have speculated that hypertriglyceridemia may impair vasodilation mechanisms, which may then increase vascular resistance and thus increase BP. High triglyceride levels lead to the formation of small dense LDL, which is particularly susceptible to oxidation to form oxidized small dense LDL. These oxidized small dense LDL particles have been demonstrated to induce endothelial dysfunction through the attenuation of endothelial nitric oxide activity and/or synthesis [32]. It is therefore possible that hypertriglyceridemia impairs vasodilation mechanisms independently of LDL. In addition, the lipolysis of triglycerides in adipose tissues has been shown to lead to non-esterified fatty acid production, which has been shown to disrupt endothelial function through the inhibition of endothelium-dependent hyperpolarization, a powerful vasodilator in small resistance arteries in both rats [33,34] and humans [35]. In addition, a retrospective longitudinal report of 1228 participants found that the baseline level of triglycerides was independently associated with a persistent increase in brachial–ankle pulse wave velocity (baPWV) and incident increases in baPWV, and that the baseline triglyceride level was a better predictor of the risk of arterial stiffness progression in healthy men than other lipid profiles [36]. However, a cohort study which included 306 persons aged ≥60 years without hypertension or cardiovascular diseases at baseline found that triglycerides were not associated with the risk of hypertension according to multivariate analysis [23]. Previous studies, including the Fenofibrate Intervention and Event Lowering in Diabetes study, have also reported a link between hypertriglyceridemia and BP [37], and that a decrease in serum triglycerides was associated with a decrease in BP [37,38,39,40].

Another interesting finding of this study was the correlation between low HDL-C and baseline hypertension and an increased risk of incident hypertension. The participants in the third HDL-C quartile (55–63 mg/dL) had a protective effect against incident hypertension. A 20-year follow-up analysis of the Chinese Community-based Cohort found independent associations between high triglycerides and low HDL-C and the risk of incident hypertension (OR 1.14, 95% CI: 1.03–1.27; OR 0.47, CI: 0.29–0.76, respectively) in 1802 subjects without hypertension [41]. In addition, a population-based cohort study of middle-aged men without baseline hypertension conducted in Finland reported a 1.6-fold (95% CI: 1.2–2.3) higher risk of new-onset hypertension for each one-standard-deviation increase in triglyceride concentration, whereas HDL-C concentration (OR = 0.7, 95% CI: 0.5–0.9) seemed to have a protective effect [28]. Various pathophysiological mechanisms have been proposed to underlie the association between the risk of new-onset hypertension and lipid levels. Patients with dyslipidemia have defective nitric oxide bioactivity and, consequently, reduced vasodilatory capacity and increased BP [42]. Furthermore, atherosclerotic lesions caused by the dysfunction of the endothelium have been associated with increased arterial stiffness and reduced arterial compliance, leading to hypertension [43]. Endothelial glycocalyx has also been demonstrated to play a key role in elasticity, inflammation, and vascular permeability, ultimately leading to cardiovascular disease. A previous study including 120 patients receiving treatment for hypertension aged >50 years investigated tertiles of HDL-C (upper tertile, ≥71 mg/dL; lower tertiles <71 mg/dL) and demonstrated that a high HDL-C level (71 to 101 mg/dL) had a moderately protective effect on endothelial glycocalyx and subsequently endothelial function [44]. In our study, those with incident hypertension had other features suggestive of metabolic syndrome, including higher body mass index and fasting glucose, older age, more frequent history of smoking and alcohol consumption, and less frequent regular exercise habits, which may have contributed to hypertension development. The observed association could also partly be explained by other confounding factors shared among these individuals, such as high dietary carbohydrate, salt, or fat intake.

In this study, we also found that high Chol/HDL-C was associated with an elevated risk of incident hypertension. In addition, participants in the second Chol/HDL-C quartile (2.97–3.54) had a 1.44-times increased risk of incident hypertension. Dyslipidemia can cause atherosclerosis, which is an important risk factor for adverse cardiovascular outcomes [15]. A study of 2103 middle-aged men suggested that compared with changes in the LDL-C/HDL-C ratio, changes in the Chol/HDL-C ratio may be associated with more substantial changes in metabolic markers, and that these changes could predict the risk of ischemic heart disease and are associated with insulin resistance [45]. A meta-analysis that included 11 studies reported a pooled adjusted relative risk of hypertension of 1.43 (95% CI 1.27–1.62) for homeostasis model assessment insulin resistance when comparing the highest and lowest categories [46]. In addition, a study of Swedish women over a 17-year period indicated that Chol/HDL-C was a better predictor than non-HDL-C, which includes very low-density lipoprotein cholesterol, intermediate lipoproteins, and LDL-C [47]. A correlation analysis of the Korean National Health and Nutrition Examination Survey 2017 reported that DBP (*r* = −0.198, *p* < 0.001) and SBP (*r* = −0.188, *p* < 0.001) were negatively correlated with the percentage of HDL-C in Chol, HDL-C/Chol (%) [48]. In addition, a retrospective longitudinal study of 1228 individuals who received repeated baPWV measurements after an interval of >3 years found an association between persistently high baPWV and incident elevated baPWV and baseline triglycerides and HDL-C, showing that they were better predictors of arterial stiffness progression in healthy men than other lipid variables [36]. Another cross-sectional study of 16,733 Chinese adults which investigated baPWV and biochemical and clinical indices reported consistent associations between Chol/HDL-C and arterial stiffness over a range of LDL-C levels, including a level of <70 mg/dL [49]. The positive association between triglycerides and HDL-C and arterial stiffness should be considered in vascular health management programs. Arterial stiffness, as measured using PWV, has been reported to be substantially higher in patients with hypertension independently of BP [50]. These findings may imply that the association between lipoproteins and atherosclerotic disease depends on the balance between atherothrombotic and atherogenic lipoproteins rather than single lipoproteins.

In this study, we found an association between high LDL-C and a lower prevalence of baseline hypertension (Table 2). As the results were cross-sectional, we could see which baseline hypertension group also had high lipid profiles, and thus may have used more lipid-lowering medications, which could have caused these results. Data on lipid-lowering medication use are lacking in the TWB, so we could not evaluate the impact of dyslipidemia on hypertension, especially in the cross-sectional study. This may partially explain these findings, which are in contrast to the previous consensus. Future studies may focus on examining whether lipid-lowering therapy can delay the development of hypertension.

This study had several strengths, including a large sample size with complete follow-up data. However, there were also some limitations. First, we lacked data on medications that may have had an effect on dyslipidemia, and this may have led to an underestimation of the association between incident hypertension and lipid profiles. Second, data on several other factors that could cause incident hypertension, such as proteinuria and daily salt intake, were also lacking. Third, our results may not be applicable to other groups, since all of our participants were of Chinese ethnicity. Finally, sample bias was possible, as only approximately 25% of the participants received follow-up evaluations.

In conclusion, we showed that high Chol/HDL-C, low HDL-C, and hypertriglyceridemia were associated with an increased risk of developing incident hypertension in a Taiwanese population. These lipid profiles are inexpensive and easy to calculate in clinical practice, and evaluating them before the onset of hypertension may help to improve the primary prevention of incident hypertension, identify patients at high risk of hypertension, and lead to the application of aggressive and individual-based treatments.

## Figures and Tables

**Table 1:** Comparisons of clinical characteristics between baseline hypertension groups (*n* = 26,965).

Characteristics	Baseline Hypertension (−) (*n* = 21,454)	Baseline Hypertension (+) (*n* = 5511)	*p*
Age (years)	49.7 ± 10.3	57.2 ± 8.3	<0.001
Male sex (%)	32.2	48.0	<0.001
DM (%)	3.5	12.2	<0.001
Smoking history (%)	23.9	32.2	<0.001
Alcohol history (%)	2.7	3.6	0.001
Regular exercise (%)	46.1	56.6	<0.001
Systolic BP (mmHg)	112.0 ± 13.0	139.3 ± 16.5	<0.001
Diastolic BP (mmHg)	69.8 ± 9.0	83.0 ± 11.0	<0.001
Body mass index (kg/m^2^)	23.6 ± 3.4	25.9 ± 3.7	<0.001
Laboratory parameters			
Fasting glucose (mg/dL)	94.5 ± 18.7	102.8 ± 24.6	<0.001
Hemoglobin (g/dL)	13.6 ± 1.6	14.2 ± 1.5	<0.001
Triglyceride (mg/dL)	107.9 ± 79.9	137.7 ± 90.5	<0.001
Total cholesterol (mg/dL)	195.1 ± 35.3	196.9 ± 35.6	<0.001
HDL-C (mg/dL)	55.2 ± 13.2	50.5 ± 12.4	<0.001
LDL-C (mg/dL)	121.3 ± 31.6	122.8 ± 31.5	0.002
Chol/HDL-C ratio	3.70 ± 1.01	4.07 ± 1.02	<0.001
eGFR (mL/min/1.73 m^2^)	111.1 ± 24.9	101.5 ± 25.7	<0.001
Uric acid (mg/dL)	5.3 ± 1.4	6.0 ± 1.5	<0.001

Abbreviations: DM, diabetes mellitus; HDL-C, high-density lipoprotein cholesterol; LDL-C, low-density lipoprotein cholesterol; Chol/HDL-C, the ratio of total cholesterol to HDL-C; eGFR, estimated glomerular filtration rate.

**Table 2:** Associations of lipid profile quartiles with baseline hypertension according to logistic regression analysis for the whole cohort (*n* = 26,965).

Lipid Profile Quartile	Univariable (Baseline Hypertension)		Multivariable (Baseline Hypertension)	
	Odds Ratio (95% CI)	*p*	Odds Ratio (95% CI)	*p*
Triglyceride				
Quartile 1	Reference		Reference	
Quartile 2	1.888 (1.726–2.066)	<0.001	1.197 (1.064–1.345)	0.003
Quartile 3	2.671 (2.449–2.912)	<0.001	1.330 (1.188–1.490)	<0.001
Quartile 4	4.042 (3.718–4.395)	<0.001	1.612 (1.440–1.804)	<0.001
Total cholesterol				
Quartile 1	Reference		Reference	
Quartile 2	1.071 (0.993–1.154)	0.074	0.958 (0.869–1.055)	0.382
Quartile 3	1.225 (1.138–1.319)	<0.001	1.032 (0.938–1.135)	0.518
Quartile 4	1.284 (1.192–1.382)	<0.001	0.860 (0.779–0.948)	0.002
HDL-C				
Quartile 1	Reference		Reference	
Quartile 2	0.698 (0.652–0.747)	<0.001	0.886 (0.794–0.943)	0.001
Quartile 3	0.530 (0.493–0.570)	<0.001	0.874 (0.795–0.962)	0.006
Quartile 4	0.388 (0.359–0.419)	<0.001	0.774 (0.696–0.862)	<0.001
LDL-C				
Quartile 1	Reference		Reference	
Quartile 2	1.044 (0.968–1.126)	0.265	0.893 (0.810–0.985)	0.023
Quartile 3	1.177 (1.093–1.268)	<0.001	0.922 (0.839–1.014)	0.096
Quartile 4	1.329 (1.234–1.431)	<0.001	0.784 (0.712–0.864)	<0.001
Chol/HDL-C ratio				
Quartile 1	Reference		Reference	
Quartile 2	1.548 (1.421–1.686)	<0.001	1.024 (0.916–1.145)	0.676
Quartile 3	2.235 (2.059–2.425)	<0.001	1.075 (0.965–1.197)	0.189
Quartile 4	2.951 (2.727–3.199)	<0.001	1.112 (0.997–1.239)	0.056

Abbreviations are the same as in Table 1. Data are shown as odds ratio and 95% confidence interval (CI). Adjusted for age, sex, diabetes, smoking and alcohol history, regular exercise habits, body mass index, uric acid, eGFR, fasting glucose, and hemoglobin. Cutoff quartile values were ≤63, 64–88, 89–127, and >127 mg/dL for triglycerides; ≤171, 172–193,194–217, and >217 mg/dL for total cholesterol; ≤46, 47–54, 55–63, and >63 mg/dL for HDL-C; ≤ 99, 100–119, 120–141, and >141 mg/dL for LDL-C; and ≤2.96, 2.97–3.54, 3.55–4.29, and >4.29 for Chol/HDL-C ratio.

**Table 3:** Comparisons of clinical characteristics between the participants with and without incident hypertension (*n* = 21,454).

Characteristics	Incident Hypertension (−) (*n* = 18,295)	Incident Hypertension (+) (*n* = 3159)	*p*
Age (years)	47.8 ± 10.2	54.8 ± 9.2	<0.001
Male sex (%)	30.2	43.3	<0.001
DM (%)	3.0	6.6	<0.001
Smoking history (%)	22.9	29.9	<0.001
Alcohol history (%)	2.5	4.2	<0.001
Regular exercise habits (%)	45.0	52.7	<0.001
Systolic BP (mmHg)	109.8 ± 12.3	124.3 ± 10.1	<0.001
Diastolic BP (mmHg)	68.7 ± 8.6	76.5 ± 8.1	<0.001
Body mass index (kg/m^2^)	23.4 ± 3.3	25.0 ± 3.4	<0.001
Laboratory parameters			
Fasting glucose (mg/dL)	93.6 ± 17.3	99.5 ± 24.9	<0.001
Hemoglobin (g/dL)	13.6 ± 1.5	14.0 ± 1.5	<0.001
Triglyceride (mg/dL)	104.2 ± 75.8	129.3 ± 97.9	<0.001
Total cholesterol (mg/dL)	194.3 ± 35.3	199.5 ± 35.5	<0.001
HDL-C (mg/dL)	55.8 ± 13.3	120.6 ± 31.4	<0.001
LDL-C (mg/dL)	120.6 ± 31.4	125.8 ± 32.6	<0.001
Chol/HDL-C ratio	3.65 ± 1.00	4.00 ± 1.05	<0.001
eGFR (mL/min/1.73 m^2^)	112.2 ± 24.8	104.7 ± 24.6	<0.001
Uric acid (mg/dL)	5.3 ± 1.3	5.8 ± 1.4	<0.001

Abbreviations are the same as in Table 1.

**Table 4:** Associations between lipid profile quartiles and incident hypertension according to logistic regression analysis (*n* = 21,454).

Quartile of Lipid Profile	Univariable (Incident Hypertension)		Multivariable (Incident Hypertension)	
	Odds Ratio (95% CI)	*p*	Odds Ratio (95% CI)	*p*
Triglyceride				
Quartile 1	Reference		Reference	
Quartile 2	1.877 (1.652–2.132)	<0.001	1.402 (1.227–1.602)	<0.001
Quartile 3	2.354 (2.078–2.666)	<0.001	1.365 (1.196–1.558)	<0.001
Quartile 4	3.393 (3.009–3.825)	<0.001	1.617 (1.415–1.848)	<0.001
Total cholesterol				
Quartile 1	Reference		Reference	
Quartile 2	1.185 (1.061–1.324)	0.003	1.021 (0.907–1.148)	0.733
Quartile 3	1.300 (1.165–1.450)	<0.001	1.007 (0.895–1.133)	0.911
Quartile 4	1.477 (1.326–1.645)	<0.001	0.974 (0.865–1.097)	0.663
HDL-C				
Quartile 1	Reference		Reference	
Quartile 2	0.792 (0.718–0.873)	<0.001	0.994 (0.895–1.105)	0.914
Quartile 3	0.595 (0.535–0.661)	<0.001	0.886 (0.788–0.996)	0.042
Quartile 4	0.471 (0.422–0.526)	<0.001	0.819 (0.721–0.930)	0.002
LDL-C				
Quartile 1	Reference		Reference	
Quartile 2	1.132 (1.011–1.267)	0.031	0.958 (0.850–1.080)	0.481
Quartile 3	1.283 (1.150–1.433)	<0.001	0.960 (0.854–1.080)	0.499
Quartile 4	1.579 (1.418–1.759)	<0.001	0.999 (0.888–1.123)	0.987
Chol/HDL-C ratio				
Quartile 1	Reference		Reference	
Quartile 2	1.547 (1.367–1.751)	<0.001	1.144 (1.005–1.302)	0.042
Quartile 3	2.076 (1.843–2.338)	<0.001	1.149 (1.011–1.306)	0.034
Quartile 4	2.745 (2.445–3.081)	<0.001	1.225 (1.075–1.396)	0.002

Abbreviations are the same as in Table 1. Values expressed as odds ratio and 95% confidence interval (CI). Adjusted for age, sex, diabetes, smoking and alcohol history, regular exercise habits, body mass index, fasting glucose, hemoglobin, eGFR, and uric acid. The cutoff values of quartiles were ≤63, 64–88, 89–127, and >127 mg/dL for triglycerides; ≤171, 172–193,194–217, and >217 mg/dL for total cholesterol; ≤46, 47–54, 55–63, and >63 mg/dL for HDL-C; ≤99, 100–119, 120–141, and >141 mg/dL for LDL-C; and ≤2.96, 2.97–3.54, 3.55–4.29, and >4.29 for Chol/HDL-C ratio.

## Data Availability

The data underlying this study are from the Taiwan Biobank. Due to restrictions placed on the data by the Personal Information Protection Act of Taiwan, the minimal data set cannot be made publicly available. Data may be available upon request to interested researchers. Please send data requests to: Szu-Chia Chen. Division of Nephrology, Department of Internal Medicine, Kaohsiung Medical University Hospital, Kaohsiung Medical University.

## Supplementary Materials

The following are available online at https://www.mdpi.com/article/10.3390/nu14163277/s1, Table S1: Clinical characteristics of the study participants classified by sex in all study participants (*n* = 26,965), Table S2: Clinical characteristics of the study participants classified by sex in study participants without baseline hypertension disease (*n* = 21,454).
