# Supplementary material for: Dyslipidemia Increases the Risk of Incident Hypertension in a Large Taiwanese Population Follow-Up Study
Source: Nutrients. 2022 Aug 10;14(16):3277. doi: 10.3390/nu14163277 (PMC9416084; doi:10.3390/nu14163277)
Supplement: Supplementary file 1 [file nutrients-14-03277-s001.zip › nutrients-1795641-supplementary.pdf]

Supplementary Table S1. Clinical characteristics of the study participants classified by sex in all study participants (n = 26,965)

Characteristics	Male (n = 9541)	Female (n = 17,424)	p
Age (year)	51.5 ± 10.9	51.0 ± 10.1	0.001
DM (%)	7.0	4.3	< 0.001
Baseline hypertension (%)	27.7	16.5	< 0.001
Smoking history (%)	58.9	7.4	< 0.001
Alcohol history (%)	7.0	0.6	< 0.001
Regular exercise habits (%)	49.2	47.8	0.022
Systolic BP (mmHg)	122.3 ± 16.4	115.0 ± 17.8	< 0.001
Diastolic BP (mmHg)	77.0 ± 10.4	70.0 ± 10.3	< 0.001
Body mass index (kg/m^2)	25.1 ± 3.3	23.5 ± 3.6	< 0.001
Laboratory parameters			
Fasting glucose (mg/dL)	99.8 ± 22.9	94.2 ± 18.5	< 0.001
Hemoglobin (g/dL)	15.0 ± 1.2	13.0 ± 1.3	< 0.001
Triglyceride (mg/dL)	133.6 ± 100.3	103.3 ± 69.6	< 0.001
Total cholesterol (mg/dL)	191.5 ± 64.5	197.6 ± 35.7	< 0.001
HDL-C (mg/dL)	48.0 ± 11.0	57.7 ± 13.0	< 0.001
LDL-C (mg/dL)	122.0 ± 31.3	121.4 ± 31.8	0.153
Chol/HDL-C ratio	4.16 ± 1.08	3.57 ± 0.93	< 0.001
eGFR (mL/min/1.73 m^2)	99.1 ± 20.9	114.7 ± 26.0	< 0.001
Uric acid (mg/dL)	6.5 ± 1.4	4.9 ± 1.1	< 0.001

Abbreviations. DM, diabetes mellitus; HDL-C, high-density lipoprotein cholesterol; LDL-C, low-density lipoprotein cholesterol; Chol/HDL-C, the ratio of total cholesterol to HDL-C; eGFR, estimated glomerular filtration rate.

Supplementary Table S2. Clinical characteristics of the study participants classified by sex in study participants without baseline hypertension disease (n = 21,454)

Characteristics	Male (n = 6898)	Female (n = 14,556)	p
Age (year)	49.6 ± 10.9	49.8 ± 10.0	0.471
DM (%)	5.0	2.9	< 0.001
Incident hypertension (%)	19.8	12.3	< 0.001
Smoking history (%)	57.8	7.9	< 0.001
Alcohol history (%)	6.9	0.7	< 0.001
Regular exercise habits (%)	46.2	46.1	0.847
Systolic BP (mmHg)	116.1 ± 11.5	110.0 ± 13.3	< 0.001
Diastolic BP (mmHg)	73.8 ± 8.2	67.9 ± 8.7	< 0.001
Body mass index (kg/m^2)	24.6 ± 3.2	23.2 ± 3.4	< 0.001
Laboratory parameters			
Fasting glucose (mg/dL)	98.2 ± 2.2	92.8 ± 16.5	< 0.001
Hemoglobin (g/dL)	15.0 ± 1.1	13.0 ± 1.3	< 0.001
Triglyceride (mg/dL)	128.6 ± 98.9	98.1 ± 67.0	< 0.001
Total cholesterol (mg/dL)	191.8 ± 34.4	196.6 ± 35.7	< 0.001
HDL-C (mg/dL)	48.6 ± 11.1	58.4 ± 13.0	< 0.001
LDL-C (mg/dL)	122.7 ± 31.4	120.7 ± 31.7	< 0.001
Chol/HDL-C ratio	4.12 ± 1.09	3.51 ± 0.92	< 0.001

eGFR (mL/min/1.73 m^2)	100.9 ± 20.0	116.0 ± 25.6	< 0.001
Uric acid (mg/dL)	6.4 ± 1.3	4.8 ± 1.1	< 0.001

Abbreviations. DM, diabetes mellitus; HDL-C, high-density lipoprotein cholesterol; LDL-C, low-density lipoprotein cholesterol; Chol/HDL-C, the ratio of total cholesterol to HDL-C; eGFR, estimated glomerular filtration rate.
